# Correction: Tanguturi et al. Discovery of Novel Delta Opioid Receptor (DOR) Inverse Agonist and Irreversible (Non-Competitive) Antagonists. *Molecules* 2021, *26*, 6693

**DOI:** 10.3390/molecules27061969

**Published:** 2022-03-18

**Authors:** Parthasaradhireddy Tanguturi, Vibha Pathak, Sixue Zhang, Omar Moukha-Chafiq, Corinne E. Augelli-Szafran, John M. Streicher

**Affiliations:** 1 Department of Pharmacology, College of Medicine, University of Arizona, Tucson, AZ 85724, USA; parthasaradhit@arizona.edu; 2 Department of Chemistry, Division of Drug Discovery, Southern Research Institute, Birmingham, AL 35205, USA; vibhabpathak@gmail.com (V.P.); szhang@southernresearch.org (S.Z.); omoukha-chafiq@southernresearch.org (O.M.-C.); caugelli-szafran@southernresearch.org (C.E.A.-S.)

## 1. Text Correction

There was an error in the original publication [1]. The compound SYK-623 was unintentionally labeled as our own, SRI-45127, and treated as our original compound in the manuscript. SYK-623 was synthesized in another study as a control group. Our mistaken claim that this compound was our own was then exacerbated due to miscommunication amongst our scientific team. No malfeasance was intended by this mistake.

Corrections have been made throughout the text; correctly labeling SRI-45127 back to SYK-623 and adjusting the text accordingly. Further corrections have been made, with clear references and comparisons between SYK-623 and our own ligands, delineating what is unique about our compounds compared to theirs. These sections include: Abstract; Introduction Paragraphs 3 and 4; Results Sections 2.1, 2.2, 2.3, and 2.5; Discussion Paragraph 3; Materials and Methods Section 4.1.10. 

**Corrected Text**

**Abstract:** The delta opioid receptor (DOR) is a crucial receptor system that regulates pain, mood, anxiety, and similar mental states. DOR agonists, such as SNC80, and DOR-neutral antagonists, such as naltrindole, were developed to investigate the DOR in vivo and as potential therapeutics for pain and depression. However, few inverse agonists and non-competitive/irreversible antagonists have been developed, and none are widely available. This leaves a gap in our pharmacological toolbox and limits our ability to investigate the biology of this receptor. Thus, we designed and synthesized the novel compounds SRI-9342 as an irreversible antagonist and SRI-45128 as an inverse agonist. These compounds were then evaluated in vitro for their binding affinity by radioligand binding, their functional activity by ^35^S-GTPγS coupling, and their cAMP accumulation in cells expressing the human DOR. Both compounds demonstrated high binding affinity and selectivity at the DOR, and both displayed their hypothesized molecular pharmacology of irreversible antagonism (SRI-9342) or inverse agonism (SRI-45128). Together, these results demonstrate that we have successfully designed new inverse agonists and irreversible antagonists of the DOR based on a novel chemical scaffold. These new compounds will provide new tools to investigate the biology of the DOR or even new potential therapeutics.

**Introduction Paragraphs 3 and 4**

By contrast, an inverse agonist does have intrinsic activity at the orthosteric site. However, unlike an agonist, an inverse agonist shifts the energy landscape to further disfavor the receptor active state and favor the inactive state. This suppresses baseline receptor activity to the extent that it is below that of the unliganded state [3]. The first such described ligand for the DOR was ICI-174864, a peptidic inverse agonist reported by Costa and Herz [7]. Later, additional DOR inverse agonists were reported, such as (+)−KF4 [8], naltrindole (NTI) derivatives [9], amide/sulfonamide substituted NTI [10], as well as other peptidic [11–13] and nonpeptidic [14–17] molecules.

Here, we report the discovery and characterization of a new DOR non-competitive antagonist (SRI-9342) and a new inverse agonist (SRI-45128). These ligands have strong selectivity for the DOR and potent functional activity in vitro. The discovery of these ligands further expands the limited pharmacological tools available to probe the DOR and could even provide potential future therapeutics.

**Results Sections 2.1, 2.2, 2.3, 2.5**

*2.1.*  *Rational Design of DOR Ligands*

The DOR ligands SRI-9342 (irreversible antagonist) and SRI-45128 (inverse agonist) were designed based on our previously published computational docking studies of the SRI-9409 scaffold core [18], as well as DOR ligands reported in the study, including SYK-623 [19]. When this core binds to the orthosteric site of DOR, the right-hand side indole moiety would face the extracellular opening of the binding pocket and be adjacent to the functionally important K214 of DOR [20], which is also a potentially reactive residue. Thus, the idea of adding a warhead to the scaffold to further increase its binding affinity to DOR was explored, which resulted in SRI-9342. The α,β-unsaturated pyridin group of SRI-9342 is supposed to be in a proper position to form a covalent bond with the nitrogen on the side chain of K214 via Michael’s addition, turning it into an irreversible DOR antagonist. On the other hand, the cyclopropyl group of the scaffold would face the bottom of the binding pocket and form hydrophobic contact with the W274 of DOR, which is an important residue responsible for the switch between agonism and antagonism [20,21]. Therefore, chemical modifications were also explored at this position, which resulted in SRI-45128 with DOR inverse agonism. Notably, SRI-45128 was distinct from parent scaffold SRI-9409 in the sense that the introduction of carbonyl next to the cyclopropyl group (inspired by SYK-623) would reduce the basicity of the neighboring nitrogen of SRI-45128, which was no longer able to form a salt-bridge with key residue D128. In addition, SRI-45128 is distinguishable from SYK-623 in the sense that the former possessed a pyridine-4-phenylchloride moiety while the latter possessed an indole moiety on the right-hand side.

*2.2.*  *Synthesis of Novel DOR Irreversible Antagonist and Inverse Agonists*

SRI-9342 (irreversible antagonist, Scheme 1) was synthesized with a 44% yield via the reaction of naltrexone hydrochloride and *trans*-4-hydrazino-2-stilbazole dihydrochloride following the same procedures previously reported [22,23]. SRI-45128 (inverse agonist, Scheme 2) was synthesized in nine steps by using the procedures reported in [24], subject to a few modifications. In our alternative route, the protecting group on the phenolic hydroxyl group of naltrexone (**1**) was changed from methyl to benzyl to achieve an overall improvement in yields. The 6-ketone group of compounds (**2**) was protected as 1,3-dioxolane, which was followed by acetylation of the 14-OH under refluxing in Ac_2_O. The cyclopropyl methyl group of the resulting acetate compound (**3**) was exchanged for a trichloroethoxycarbonyl group at 140 °C with an excess amount of trichloroethoxycarbonyl chloride to afford carbamate (**4**). The carbamate and the acetate group in compound (**4**) were further hydrolyzed with aqueous KOH at 110 °C to afford compound (**5**). The reaction of compound (**5**) with cyclopropyl carbonyl chloride in the presence of Et_3_N afforded compound (**6**) with an 89% yield. Removal of the 1,3-dioxolane group in amide (**6**) was done by using HCl and MeOH at reflux conditions to afford ketone (**7**). Finally, further deprotection of the benzyl group in (**7**), followed by annulation with 2-(4-chlorophenyl)-3-hydroxy-prop-2-enal in the presence of ammonium acetate, afforded SRI-45128 in two steps. We also synthesized SYK-623 for use as a control group (inverse agonist, Scheme 3). This was achieved in two steps, from intermediate (**7**), by using the procedure reported in [19] with the following modifications: Compound (**7**) was reacted with phenyl hydrazine in acetic acid under reflux conditions followed by deprotection of the benzyl group to afford SYK-623. All compounds were confirmed for identity and high purity, which is sufficient for pharmacological characterization (see Methods).

*2.3.*  *All Compounds Display High DOR Binding Affinity*

All synthesized compounds were evaluated for binding to the human DOR using competition radioligand binding. All compounds showed one site full competition, suggesting full occupancy of the orthosteric binding site (Figure 1). Notably, the compounds are also bound to the DOR with high affinity with K_I_ values of 4.9–24 nM (Figure 1). We also tested SRI-45128 and SYK-623 for binding to the MOR and KOR, which would provide insight into compound selectivity. The compounds bound very weakly to the MOR, showing incomplete curves even at 10 μM. This suggests that both compounds are at least 120-fold selective for DOR over MOR. The compounds bound slightly better to the KOR, providing near-complete curves and K_I_ values, ranging from 2000 to 2500 nM, suggesting that both compounds are at least 104-fold selective for DOR over KOR (Figure 1). These results demonstrate that these compounds are high-affinity DOR ligands, and both inverse agonists display strong DOR selectivity. The expected performance of SYK-623 further confirms our findings.

*2.5.*  *SRI-45128 Displays DOR Inverse Agonism*

Similar to SRI-9342, we sought to evaluate the inverse agonist functional activity of SRI-45128 with SYK-623 as a comparison control. The GTPγS assay used above has a generally low baseline receptor activity level, at least for the opioid receptors, so we switched assays to a live cell cAMP accumulation assay. This assay uses forskolin to stimulate cAMP levels, which are then inhibited/suppressed by the Gα_I_-coupled activity of the DOR. As expected, SNC80 demonstrated potent and efficacious suppression of cAMP levels in DOR-CHO cells, which is in line with the expected activity of the receptor (Figure 3). By contrast, both SRI-45128 and SYK-623 showed efficacious inverse agonist activity and actually boosted cAMP levels, which is consistent with suppressing the baseline activity of the DOR (Figure 3). This activity was also efficacious, with an E_MAX_ of −67% and −56%, respectively. These results suggest that SRI-45128 is a robust inverse agonist, further confirmed by the performance of the SYK-623 comparison control.

**Discussion Paragraph 3**

Future works should also investigate these compounds in greater detail. Based on our binding studies, it is clear that all compounds bind selectively and with a high affinity to the DOR orthosteric site. However, the functional studies were not quite as clear. SRI-9342 displayed clear signs of irreversible antagonism at 0.1 and 1 nM. However, 10 nM caused a rapid loss of potency that continued at 100 nM and 1000 nM. The compound could be a full irreversible antagonist, and the activity at 10 nM could represent a rapid loss of receptors from the system that would eventually lead to the same reduction in potency as with other irreversible antagonists. Alternatively, the compound could have mixed activity, with irreversible antagonism at low concentrations and different functional activity, similar to competitive antagonism, at high concentrations. This mixed activity has been observed with other compounds, such as naloxonazine [30]. SRI-45128 also displayed clear inverse agonist activity. However, the potency of this activity was considerably less than the binding affinity. This could represent the poor intrinsic efficacy of the compounds, or it could represent a relatively insensitive system for baseline receptor suppression. These details should be investigated for these compounds, working out the exact mechanisms of action and activity in different DOR-related signaling systems (e.g., ERK-MAPK activation instead of cAMP signaling). In addition, all compounds should be investigated for in vivo activity and whether the in vivo testing matches the predictions made via the in vitro testing reported here.

**Materials and Methods Section 4.1.10**

4.1.10. Cyclopropyl((4bS,8R,8aS,14bR)-1,8a-dihydroxy-5,6,8a,9,14,14b-hexahydro-4,8-methanobenzofuro[2,3−a]pyrido [4,3−b]carbazol-7(8H)-yl)methanone (SYK-623)

This compound was prepared by a modification of the reported protocol [19]. A solution of compound (**7**) (200 mg, 0.45 mmol) in acetic acid (5 mL) was supplemented with phenylhydrazine (58.3 mg, 0.54 mmol), and the reaction mixture was refluxed for 2 h. After cooling to room temperature, the reaction mixture was concentrated and co-evaporated with toluene to afford a crude product. Saturated Na_2_CO_3_ was added, and the mixture was extracted with methylene chloride, dried over sodium sulfate, concentrated, chromatographed on an Isco Combiflash system using 0–5% MeOH in DCM to give a white solid which was dissolved in methanol (10 mL), and then 10% palladium black (24.6 mg, 0.02 mmol) was added to the reaction. The mixture was stirred under H_2_ atmosphere (balloon) for 2 h at room temperature and filtered through celite. The celite pad was washed with MeOH (20 mL), and the filtrate was evaporated to give a white residue, which was purified by column chromatography using 0–10% MeOH in DCM to afford SYK-623 (50 mg, 48%) as a white solid. TLC (10% MeOH/DCM): R*_f_ =* 0.35; ^1^H NMR (400 MHz, CD_3_OD) δ 7.36 (ddt, *J =* 7.8, 6.7, 1.0 Hz, ^1^H), 7.30 (ddt, *J =* 8.2, 4.1, 0.9 Hz, ^1^H), 7.07 (dddd, *J* = 8.2, 7.0, 3.8, 1.2 Hz, ^1^H), 6.94 (dddd, *J* = 8.0, 7.0, 4.7, 1.0 Hz, ^1^H), 6.63–6.51 (m, ^2^H), 5.61 (s, ^0.5^H), 5.07 (d, *J* = 6.6 Hz, ^0.5^H), 4.75 (d, *J* = 6.6 Hz, ^0.5^H), 4.49–4.38 (m, ^0.5^H), 4.18–4.08 (m, ^0.5^H), 3.44 (dd, *J =* 18.5, 6.7 Hz, ^0.5^H), 3.33–3.25 (m, ^1^H), 3.15 (td, *J* = 13.4, 3.8 Hz, ^0.5^H), 3.00–2.58 (m, ^4^H), 2.51 (td, *J* = 12.8, 5.2 Hz, ^0.5^H), 2.07 (tt, *J* = 7.9, 4.8 Hz, ^0.5^H), 1.96–1.88 (m, ^0.5^H), 1.71–1.62 (m, ^1^H), 1.00–0.90 (m, ^1^H), 0.89–0.74 (m, ^3^H). HRMS (ESI) *m*/*z* calcd for C_26_H_24_N_2_O_4_ [M + H]^+^: 429.1810, found: 429.1801; HPLC (system 1) *t*_R_ = 10.7 min, purity = 96%.

## 2. Figure/Table Legend

In the original publication, there was a labeling mistake as explained above in the legends for Figures 1–3. SYK-623 was labeled as SRI-45127. The correct legends appear below, in which the labeling has been changed from SRI-45127 to SYK-623.

**Figure 1.** Novel compounds bound to the DOR with high affinity. Compounds were tested as concentration curves competing with a fixed concentration of ^3^H-diprenorphine (see Methods). N = 3 independent replicates performed, with summary curves shown for each set; affinity (K_I_) was calculated separately for each experiment and then reported as the mean ± SEM. Experimental or reference compounds (naloxone for MOR and DOR, U50-488 for KOR) were tested at each receptor, DOR, MOR, or KOR, as noted. Reference compounds displayed expected affinities, validating the experiment, while all compounds demonstrated high affinities at the DOR. SRI-45128 and SYK-623 showed poor affinity at MOR and KOR, suggesting strong DOR selectivity.
**Figure 2.** SRI-9342 displayed DOR irreversible antagonism at low concentrations. SNC80 is a full DOR agonist, which was used to perform multiple full concentration curves using the ^35^S-GTPγS assay (see Methods). Increasingly large fixed concentrations of SRI-9342 were included in subsequent SNC80 curves. N = 3 independent experiments performed, with summary curves shown. Potency (EC_50_) and efficacy (E_MAX_) were calculated separately for each experiment and reported as the mean ± SEM. SRI-9342 caused increasing loss of efficacy at 0.1 and 1 nM without decreasing potency (which was actually higher than SNC80 alone), suggesting irreversible antagonism. Beginning at 10 nM, potency loss was observed, which could indicate mixed activity or a very strong loss of receptors from the receptor pool due to irreversible antagonist activity.
**Figure 3.** SRI-45128 caused DOR inverse agonist activity. SRI-45128, positive control SYK-623, and SNC80 reference compounds were used to modulate the cAMP accumulation caused by forskolin treatment (see Methods). One independent experiment in triplicate was performed, and the potency (EC_50_) and efficacy (E_MAX_) were reported as the derived value with 95% confidence intervals (in parentheses). As expected, SNC80 caused high potency and efficacy cAMP inhibition, which is consistent with DOR agonist activity. SRI-45128 and SYK-623 caused efficacious cAMP increases, which is contrary to DOR agonism and consistent with DOR inverse agonism.

## 3. Error in Figure/Table

In the original publication, there was a mistake in Scheme 3, Figure 1, and Figure 3 as published. As above, SYK-623 was mislabeled as SRI-45127. The corrected Scheme/Figures appear below, in which the labeling has been corrected.

**Scheme 3:**
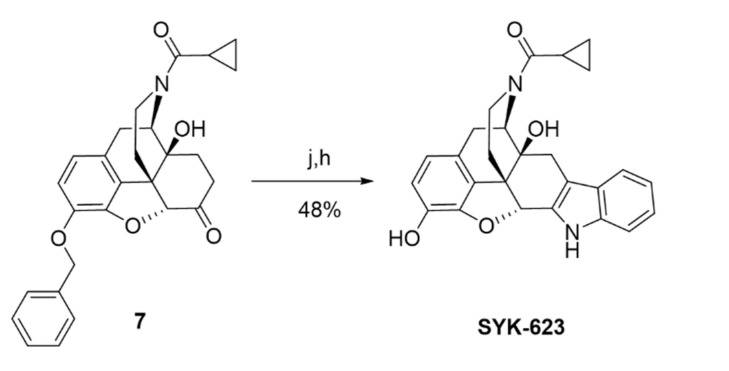
Reagents and conditions: (j) phenyl hydrazine, AcOH, reflux; (h) 10% Pd/C, H_2_, MeOH.

**Figure 1:**
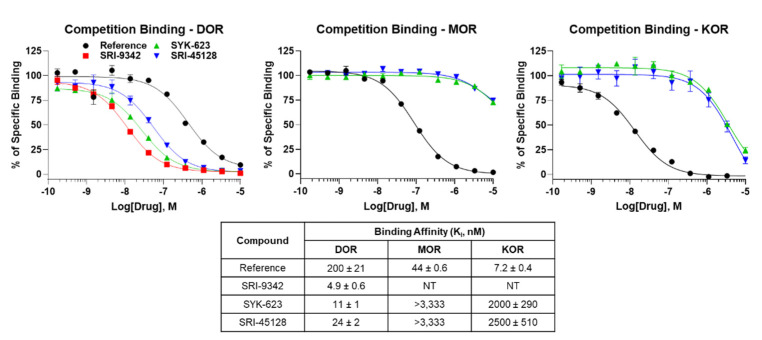
Novel compounds bound to the DOR with high affinity. Compounds were tested as concentration curves competing with a fixed concentration of ^3^H-diprenorphine (see Methods). N = 3 independent replicates performed, with summary curves shown for each set; affinity (K_I_) was calculated separately for each experiment and then reported as the mean ± SEM. Experimental or reference compounds (naloxone for MOR and DOR, U50-488 for KOR) were tested at each receptor, DOR, MOR, or KOR, as noted. Reference compounds displayed expected affinities, validating the experiment, while all compounds demonstrated high affinities at the DOR. SRI-45128 and SYK-623 showed poor affinity at MOR and KOR, suggesting strong DOR selectivity.

**Figure 3:**
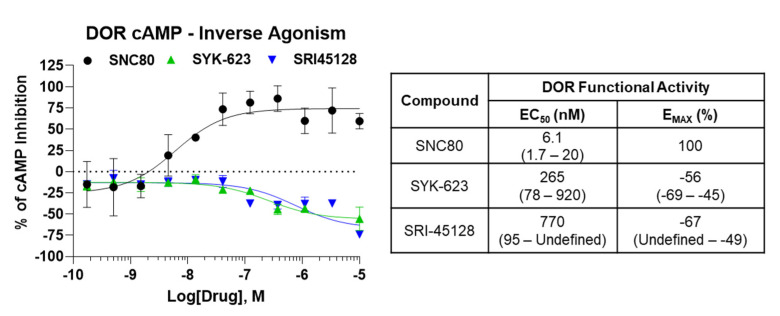
SRI-45128 caused DOR inverse agonist activity. SRI-45128, positive control SYK-623, and SNC80 reference compounds were used to modulate the cAMP accumulation caused by forskolin treatment (see Methods). One independent experiment in triplicate was performed, and the potency (EC_50_) and efficacy (E_MAX_) were reported as the derived value with 95% confidence intervals (in parentheses). As expected, SNC80 caused high potency and efficacy cAMP inhibition, which is consistent with DOR agonist activity. SRI-45128 and SYK-623 caused efficacious cAMP increases, which is contrary to DOR agonism and consistent with DOR inverse agonism.

## 4. Errors in References

In the original publication, the Reference [6] has been updated as [25]; the Reference [21] has been updated as [19]. Besides, due to adding new References, the numeration of References with respect to the original publication has been modified. References [2,3,4,5] will appear in the document as References [6,8–10] respectively.

The authors apologize for any inconvenience caused and state that the scientific conclusions are unaffected. The original publication has also been updated.
